# Communication quality shapes the sustainability of pediatric early warning system

**DOI:** 10.1186/s12913-026-15265-9

**Published:** 2026-08-01

**Authors:** Sara Malone, Luke Zabotka, Rebeca Caires, Veronica Easson, Samantha Hayes, Tessa Concepcion, Miguel Aguilar Barragan, Adolfo Cardenas, Bobbi J. Carothers, Jocelyn Rivera, Ana Berenice Aguilar Román, Andreia Ribeiro Pereira Aguiar de Paula, Carolina Abarzua, Judith Cornejo Barrera, Lupe Nataly Mora Robles, María Sánchez-Martín, Norma Araceli López Facundo, Rosdali Diaz Coronado, Veronica Soto Chavez, Charles Goss, Douglas A. Luke, Dylan E. Graetz, Virginia McKay, Asya Agulnik

**Affiliations:** 1 Bursky School of Public Health, Washington University in St. Louis, St. Louis, MO USA; 2 Department of Global Pediatric Medicine, St. Jude Children’s Research Hospital, Memphis, TN USA; 3 Hospital Infantil De Oncologia, Querétaro, Mexico; 4 School of Medicine, Washington University in St. Louis, St. Louis, MO USA; 5 Hospital General de Zona 2 IMSS Tapachula ONCOCREAN, Tapachula, Mexico; 6 Hospital de Cancer Infanto Juvenil de Barretos, Barretos, Brasil; 7 Hospital Hernan Henriquez Aravena, Temuco, Chile; 8 Hospital Infantil De Tamaulipas, Ciudad Victoria, Tamaulipas, Mexico; 9 Hospital De Solca Cuenca, Cuenca, Ecuador; 10 Hospital La Paz, Madrid, Spain; 11 Hospital Materno Infantil del ISSEMYM, Toluca, Mexico; 12 Instituto Nacional de Enfermedades Neoplásicas, Lima, Peru; 13 Hospital Civil de Guadalajara “Dr. Juan I. Menchaca”, Guadalajara, Mexico

**Keywords:** Pediatric early warning systems, Pediatric oncology, Pediatrics, Communication quality, Sustainability capacity

## Abstract

**Background:**

Pediatric early warning systems (PEWS) are quality improvement interventions that reduce preventable mortality during childhood cancer treatment, but sustaining PEWS implementation remains challenging. Although effective team communication has been linked to improved hospital care delivery, its role in supporting long-term PEWS sustainability is not well understood. This study examined the relationship between team communication quality and PEWS sustainability capacity at resource-diverse hospitals providing childhood cancer care.

**Methods:**

A cross-sectional survey was conducted among clinicians involved in childhood cancer care and PEWS use at 47 hospitals in Latin America and Spain participating in Proyecto EVAT, a quality improvement collaborative focused on PEWS implementation and sustainability. Team communication quality was assessed using the validated CritCom instrument, and sustainability capacity was measured using the Clinical Sustainability Assessment Tool (CSAT). Associations between communication quality and sustainability capacity were evaluated using Spearman’s correlation and a linear mixed-effects model accounting for hospital-level clustering.

**Results:**

A total of 1,288 clinicians participated. Communication quality was positively associated with sustainability capacity (Spearman’s ρ = 0.58). In adjusted analyses, each one-unit increase in CritCom score was associated with a 0.50-unit increase in CSAT score (*p* < 0.001). Physicians and clinicians in PEWS leadership roles reported higher communication quality and sustainability capacity than nurses and non-leadership staff. Respondents working in intensive care and non-clinical settings reported lower sustainability capacity than those working on inpatient wards. Hospital-level characteristics, including funding type and childhood cancer patient volume, were not associated with sustainability capacity.

**Conclusions:**

Higher-quality team communication independently predicts greater capacity to sustain PEWS across resource-diverse hospitals providing childhood cancer care. Differences by professional role and clinical setting suggest that targeted communication strategies may strengthen the long-term sustainability of evidence-based practices and improve global childhood cancer care outcomes.

**Supplementary Information:**

The online version contains supplementary material available at https://doi.org/10.1186/s12913-026-15265-9.

## Introduction

Children with cancer experience repeated hospitalizations throughout the trajectory of their illness and treatment, each representing a discrete period of heightened clinical vulnerability. During these admissions, the ability of care teams to recognize clinical deterioration early and respond in a coordinated, timely manner is critical to patient survival. Pediatric early warning systems (PEWS) have emerged as effective interventions to support early recognition of deterioration, with evidence demonstrating reduction in inpatient mortality when implemented into routine care [1–3]. PEWS function by standardizing routine clinical assessment for hospitalized patients and escalation of care for those with deterioration. PEWS assessments are often conducted by nurses as part of routine vital sign assessment, however, appropriate escalation of care and subsequent effectiveness depends on consistent team engagement across clinical roles and inpatient units, which both leverage and further reinforce communication between different healthcare staff providing care [2, 4].

While PEWS implementation improves patient outcomes, implementation alone is insufficient for long-term impact [3, 5]. Studies of PEWS in resource-variable hospitals have demonstrated feasible implementation and mortality benefit, yet sustainment remains challenging and context-dependent, with ongoing patient benefits contingent on continued delivery of high-quality PEWS use following initial implementation [3, 6]. Additionally, work has highlighted that perceived communication quality during PEWS delivery varies meaningfully by professional role and clinical setting, suggesting that team-level dynamics shape how PEWS are used and sustained in clinical practice [1, 7].

Existing sustainability frameworks provide a structural lens to understand intervention sustainment, known as the ongoing delivery of the program or practice in routine care after implementation. However, these do not adequately capture the interpersonal and communicative processes that define real-world multidisciplinary clinical teams, particularly in the care of complex patients, such as children with cancer [8, 9]. In busy care settings, the delivery of PEWS depends not only on whether staff know the tool, but on whether teams can reliably communicate across clinical roles and hospital units. Prior work on psychological safety, shared mental models, and handoff processes in acute care demonstrates that if communication breaks down, early warning signs go unrecognized, escalation is delayed, and the clinical value of interventions like PEWS is lost. This gap is especially consequential in global oncology settings, where resource heterogeneity amplifies the role of team communication as both a mechanism for sustained care delivery and a potential barrier to it [10]. In lower-resource contexts, higher staffing ratios, higher patient volumes, and less formalized team structures place greater demands on the quality of interpersonal communication to compensate for resource limitations.

Understanding how communication quality relates to sustainability capacity is essential to inform implementation strategies that promote maintained use of evidence-based interventions across diverse-resource contexts. The relationship between communication quality and the capacity to sustain interventions like PEWS over time, however, has not been formally examined. This study examines the relationship between perceptions of team communication quality and sustainability capacity among clinicians using PEWS across resource-variable hospitals providing childhood cancer care in Latin America and Spain.

## Methods

This study employed a cross-sectional, observational survey design to examine communication quality and sustainability capacity across hospitals using PEWS. This work was conducted as part of a larger longitudinal study to explore determinates to PEWS sustainability [11]. We followed the STROBE statement for reporting observational studies.

### Settings

Proyecto Escala de Valoracion de Alerta Temprana (EVAT) is a quality improvement collaborative started in 2017 that aims to improve hospital outcomes for children undergoing cancer treatment in Latin America and Spain by supporting implementation of a Spanish- or Portuguese-language PEWS. The project is organized by St. Jude Children’s Research Hospital in collaboration with regional partners and leverages implementation facilitation to support local teams implementing PEWS [12]. Any hospital providing childhood care with staff who speak Spanish or Portuguese is eligible join. Proyecto EVAT hospitals range in resources to provide childhood cancer care, with most self-identifying as having resource limitations due to a broad range of challenges, including inadequate nursing and physician staffing to identify and manage clinical deterioration, limited ICU space to accommodate children with cancer, and patients with low socioeconomic, educational, and nutritional indicators [13–15]. Hospitals that participate comprise both standalone pediatric hospitals and hospitals with a dedicated pediatric unit within a larger medical center, all of which were included in this analysis.

Hospitals enroll in Proyecto EVAT in annual cohorts of approximately 10–20 hospitals and are led through a standardized implementation strategy including training, virtual facilitation, and mentorship by the St. Jude and regional expert team. The Proyecto EVAT implementations strategy has been described previously [11, 12]. Briefly, hospitals are guided through a 3-phase implementation process including planning, implementation, and sustainability via bimonthly virtual mentorship meetings. Successful PEWS implementation is defined as correct PEWS use with fidelity measured as 3 types of PEWS errors (omissions, calculation errors, and algorithm non-adherence). After successful implementation, hospitals continue collaborating with Proyecto EVAT through monthly virtual meetings and/or as mentor centers but receive no further PEWS implementation support. To date, 122 hospitals providing childhood cancer care in 20 countries participate in Proyecto EVAT. Of these, 69 have successfully implemented PEWS; the remainder are in the process of implementation.

### Participants

All hospitals participating in Proyecto EVAT who successfully completed PEWS, defined as above, before April 2025 (*n* = 53) were invited to participate. Of these, 47 (88.6%) agreed to participate. At each participating hospital, local site leads identified staff caring for hospitalized children with cancer and using PEWS. Participants included physicians, nurses, research team members, data managers, and other clinical staff. Eligible staff were invited to participate in the anonymous electronic study survey.

### Procedures

This study was approved by the St. Jude Children’s Research Hospital Institutional Review Board (IRB # 22–0975) as exempt by Category 2. The Spanish or Portuguese language survey was administered via Qualtrics and distributed through anonymous email links. The survey consisted of three broad sections: participant demographics, a measure of communication quality, and a measure of capacity for PEWS sustainment. Participants were given one month to complete the survey, and 3 follow-up reminders were distributed at 7-day intervals. Data collection was conducted between April and May 2025, and the survey took about 20 min to complete. No formal consent was required; completion of the survey was deemed as agreement to participate in the study. Additionally, hospital characteristics were collected from local site leads annually as part of the parent study.

### Measures

#### Communication quality

Communication quality was assessed using CritCom, a validated instrument with high internal consistency (α = 0.97) that has been previously developed and validated in Spanish, Portuguese, and other languages [16]. CritCom (Supplement A) describes the quality of team communication across six domains: actionability, clarity, tone, empowerment, collaboration, and leadership. Each domain contains five items rated on a five-point Likert scale (1 = “almost never” to 5 = “almost always”), with higher scores representing better communication. Domain and composite scores are computed as item averages.

#### Sustainability capacity

Clinical sustainability capacity was measured using the Clinical Sustainability Assessment Tool (CSAT), previously translated and validated within Proyecto EVAT [5] in Spanish and Portuguese (Cronbach’s α = 0.98). CSAT (Supplement B) assesses seven domains: staff engagement, partner engagement, organizational readiness, workflow integration, implementation and training, monitoring and evaluation, and outcomes and effectiveness. Each domain contains five items rated on a five-point Likert scale (1 = “strongly disagree” to 5 = “strongly agree”), with higher scores representing greater sustainability capacity. Scores are averaged to yield domain and overall scores.

#### Demographics and intervention characteristics

The survey included participant demographics, such as hospital, profession, primary clinical unit, years of experience, age, gender, and role in PEWS. All demographic variables were captured categorically. Intervention-specific questions assessed delivery frequency, perceived importance of the intervention, and perceived implementation difficulty.

#### Hospital characteristics

For participating hospitals, local site leads completed an annual survey that described hospital characteristics, including hospital type, funding structure, intensive care unit availability, and the structure and staffing of pediatric oncology (i.e. nurse ratio, annual new diagnoses).

### Analyses

All analyses were conducted in R version 4.5.3, using lme4 for mixed effects modeling [17]. First, we calculated descriptive statistics to summarize participant and hospital demographics. Composite scores for CritCom and CSAT were computed as overall item averages for each participant. Between-group differences in CritCom and CSAT scores across demographic and professional categories were examined using bivariate analyses (Kruskal-Wallis, Dunn’s post hoc with Bonferroni correction), and the association between CritCom and CSAT scores was quantified using Spearman’s rank correlation. Results were considered statistically significant when the p value was less than 0.05 (*p* < 0.05).

To examine the independent relationship between communication quality and sustainability capacity while accounting for hospital-level clustering, we used a generalized linear multilevel model. Sustainability capacity score (continuous) was the dependent variable, with communication quality as the primary independent variable of interest. Covariates were selected based on hypothesized associations informed by prior modeling work; fixed effects were assigned to communication quality score, perceived importance of delivering high-quality care, profession, role within intervention implementation, gender, and primary hospital unit. A one-level random effects structure was specified at the hospital level. Models were fit using restricted maximum likelihood (REML).

## Results

A total of 1,930 participants across 47 hospitals were approached; 1,288 completed the survey and were included in this analysis (response rate: 63.6%, Table 1). Respondents were predominantly clinicians providing direct patient care: 59.1% identified as nursing professionals and 32.9% as physicians or physician trainees, while non-clinical staff such as data managers and research staff accounted for less than 1% of respondents. Over 90% of respondents reported working in a clinical area of their hospital, with the most common unit being a pediatric or hematology-oncology ward (83.7%) followed by the ICU (8.2%). Regarding institutional tenure, the largest proportion of respondents reported having been at their current institution for fewer than 5 years (39.8%). Full demographic characteristics are presented in Table 1. Most respondents worked at hospitals in Mexico (*n* = 458, 37.3%), followed by Brazil, Peru, Chile, and Colombia. Participating hospitals completed PEWS implementation between 2014 and 2024 and included diverse settings providing childhood cancer care in the region, with most having public funding structure (37/47, 79.0%; Table 2). Full hospital characteristics are presented in Table 2.

**Table 1:** Participant demographics

	*N* = 1,228^*1*^
**Profession**	
Nurse or Nurse Technician	726 (59.1%)
Physician or Physician Trainee	404 (32.9%)
Healthcare Administration	34 (2.8%)
Other	56 (4.6%)
Data Manager/Research	8 (0.7%)
**Clinical work location**	
Pediatric or Hem-Onc Bone Transplant Ward	1,028 (83.7%)
Intensive Care Unit	101 (8.2%)
Non-Clinical	26 (2.1%)
Emergency Department	22 (1.8%)
Other	51 (4.2%)
**Role in PEWS**	
PEWS implementation leader	216 (17.6%)
Clinical staff	936 (76.2%)
Hospital administrator	19 (1.5%)
Data manager	14 (1.1%)
Other	42 (3.5%)
**Years of experience**	
<1	94 (6.8%)
1–5	448 (33%)
6–10	279 (20%)
11–15	248 (18%)
16–20	148 (11%)
20+	157 (11%)
**Gender**	
Male	205 (15%)
Female	1,164 (85%)
Other	5 (0.4%)

^*1*^ n (%)

**Table 2:** Hospital characteristics

	*N* = 47^*1*^
**Country**	
Argentina	2 (4.3%)
Brazil	4 (8.5%)
Chile	2 (4.3%)
Colombia	2 (4.3%)
Dominican Republic	2 (4.3%)
Ecuador	3 (6.4%)
El Salvador	1 (2.1%)
Guatemala	1 (2.1%)
Honduras	1 (2.1%)
Mexico	21 (45%)
Panama	2 (4.3%)
Paraguay	1 (2.1%)
Peru	4 (8.5%)
Spain	1 (2.1%)
**World bank income group**	
High income	5 (11%)
Lower middle income	1 (2.1%)
Upper middle income	41 (87%)
**Funding structure**	
Mix (Public/private partnership)	6 (13%)
Private (foundation or similar)	4 (8.5%)
Public (government)	37 (79%)
**Organization type**	
General Hospital (Adults And Pediatric Patients)	19 (40%)
Oncology Hospital (Adults And Pediatric Oncology Patients)	7 (15%)
Pediatric Multidiciplinary Hospital	17 (36%)
Pediatric Oncology Hospital	2 (4.3%)
Women’s and Children’s Hospital	2 (4.3%)
**Number of inpatient beds**	187 (120, 400)
**Number of annual new oncologic diagnoses**	71 (35, 150)
**ICU Type**	
Adult ICU with pediatric beds	4 (8.5%)
Intermediate Care Unit	1 (2.1%)
Pediatric Oncology ICU	7 (15%)
Pedaitric ICU	34 (72%)
ICU Patient care on the wards	1 (2.1%)
**ICU Bed Number**	10 (6, 14)
**Nurse Staffing Ratio**	5 (4, 6)
**Implementation Completion Year**	
2014	1 (2.1%)
2016	1 (2.1%)
2018	9 (19%)
2019	8 (17%)
2020	12 (26%)
2021	3 (6.4%)
2022	5 (11%)
2023	5 (11%)
2024	3 (6.4%)

^*1*^ n (%); Median (Q1, Q3)

The overall median communication quality (CritCom) score among participants was 4.13 (IQR: 3.83–4.67). There were statistically significant differences in CritCom scores across participants with different professions and roles in PEWS implementation (Table 3). Respondents in leadership (administrative, management) positions reported significantly higher CritCom scores (4.51, IQR: 4.05, 4.86) than both clinical staff (4.26, IQR: 3.94, 4.74]) and those in other roles (3.91, IQR: 3.66, 4.41). Younger respondents also reported higher communication quality: those aged 30 and below scored significantly higher (4.46, IQR: 4.00, 4.89) than respondents in both the 40–49 (4.23, IQR: 3.89, 4.71) and 30–39 (4.26, IQR: 3.97, 4.74) age groups. The Dunn’s test showed that within professional roles, physicians scored communication quality significantly higher than nurses (4.37, IQR: 3.93, 4.83 vs. 4.03, IQR: 3.80, 4.53), and healthcare administrators scored significantly higher than nurses (4.60, IQR: 3.97, 4.83).

**Table 3:** Distribution of median CSAT and CritCom scores across demographic variables of participants

	CSAT		CritCom	
	Median (IQR)	*p* ^1^	Median (IQR)	*p* ^1^
**Profession**		< 0.001		< 0.001
Nurse or Nurse Tech	4.04 (IQR: 3.80–4.52)		4.17 (IQR: 3.91–4.69)	
Physician or Physician Trainee	4.40 (IQR: 3.93–4.83)		4.46 (IQR: 4.00–4.86)	
Healthcare Administration	4.48 (IQR: 3.88–4.88)		4.54 (IQR: 4.19–4.89)	
Other	4.07 (IQR: 3.86–4.50)		4.20 (IQR: 3.89–4.68)	
Data Manager/Research	4.00 (IQR: 3.81–4.09)		4.41 (IQR: 3.93–4.68)	
**Clinical work location**		0.057		0.225
Pediatric or Hem-Onc Ward	4.10 (IQR: 3.83–4.67)		4.26 (IQR: 3.94–4.77)	
ICU	4.37 (IQR: 3.90–4.77)		4.34 (IQR: 4.03–4.77)	
Non-Clinical	4.27 (IQR: 4.00–4.76)		4.51 (IQR: 3.99–4.86)	
Other	4.07 (IQR: 3.85–4.50)		4.26 (IQR: 3.87–4.70)	
ED	4.23 (IQR: 3.80–4.92)		4.60 (IQR: 4.01–4.94)	
**Role in PEWS**		< 0.001		< 0.001
PEWS implementation leader	4.37 (IQR: 3.96–4.80)		4.51 (IQR: 4.05–4.86)	
Clinical staff	4.10 (IQR: 3.83–4.63)		4.26 (IQR: 3.94–4.74)	
Hospital administrator	4.27 (IQR: 3.77–4.88)		4.20 (IQR: 3.86–4.94)	
Data manager	3.97 (IQR: 3.54–4.00)		4.17 (IQR: 3.61–4.57)	
Other	3.87 (IQR: 3.42–4.37)		3.91 (IQR: 3.66–4.41)	
**Years of experience**		0.234		0.068
< 1	4.10 (IQR: 3.78–4.77)		4.39 (IQR: 3.97–4.85)	
1–5	4.15 (IQR: 3.87–4.77)		4.34 (IQR: 3.97–4.83)	
6–10	4.08 (IQR: 3.83–4.53)		4.20 (IQR: 3.97–4.68)	
11–15	4.10 (IQR: 3.83–4.63)		4.26 (IQR: 3.93–4.73)	
16–20	4.10 (IQR: 3.80–4.60)		4.26 (IQR: 3.85–4.74)	
20+	4.27 (IQR: 3.90–4.69)		4.24 (IQR: 3.91–4.71)	
**Age**		0.013		0.003
< 30	4.23 (IQR: 3.80–4.83)		4.46 (IQR: 4.00–4.89)	
30–39	4.07 (IQR: 3.87–4.60)		4.26 (IQR: 3.97–4.74)	
40–49	4.07 (IQR: 3.80–4.62)		4.23 (IQR: 3.89–4.71)	
50+	4.32 (IQR: 3.97–4.72)		4.26 (IQR: 3.98–4.71)	
**Gender**		0.350		0.599
Male	4.15 (IQR: 3.87–4.73)		4.33 (IQR: 4.00–4.77)	
Female	4.13 (IQR: 3.83–4.67)		4.26 (IQR: 3.94–4.74)	
Other	4.00 (IQR: 3.33-4.00)		4.74 (IQR: 4.40–4.74)	

^1^ Kruskal-Wallis test

The median sustainability capacity (CSAT) score among participants was 4.25 (IQR: 3.93–4.73, Table 3). We observed similar patterns in group-level differences in CSAT scores as the CritCom score. Respondents in leadership roles reported significantly higher clinical capacity for sustainability than both clinical staff and those in other roles (4.43, IQR: 4.03, 4.83 vs. 4.23, IQR: 3.90, 4.70 and 3.83, IQR: 3.41, 4.38, respectively). In contrast to the CritCom pattern by participant age, respondents aged 30 and under reported significantly higher clinical capacity (4.43 [4.00, 4.87]) than those aged 30–39 (4.23, IQR: 3.93, 4.70) and 40–49 (4.20, IQR: 3.83, 4.70). By profession, physicians reported significantly higher clinical capacity than nurses (4.46, IQR: 4.00, 4.86 vs. 4.17, IQR: 3.91, 4.69); Dunn’s test showed that the difference between healthcare administrators and nurses did not reach statistical significance.

**Fig. 1:**
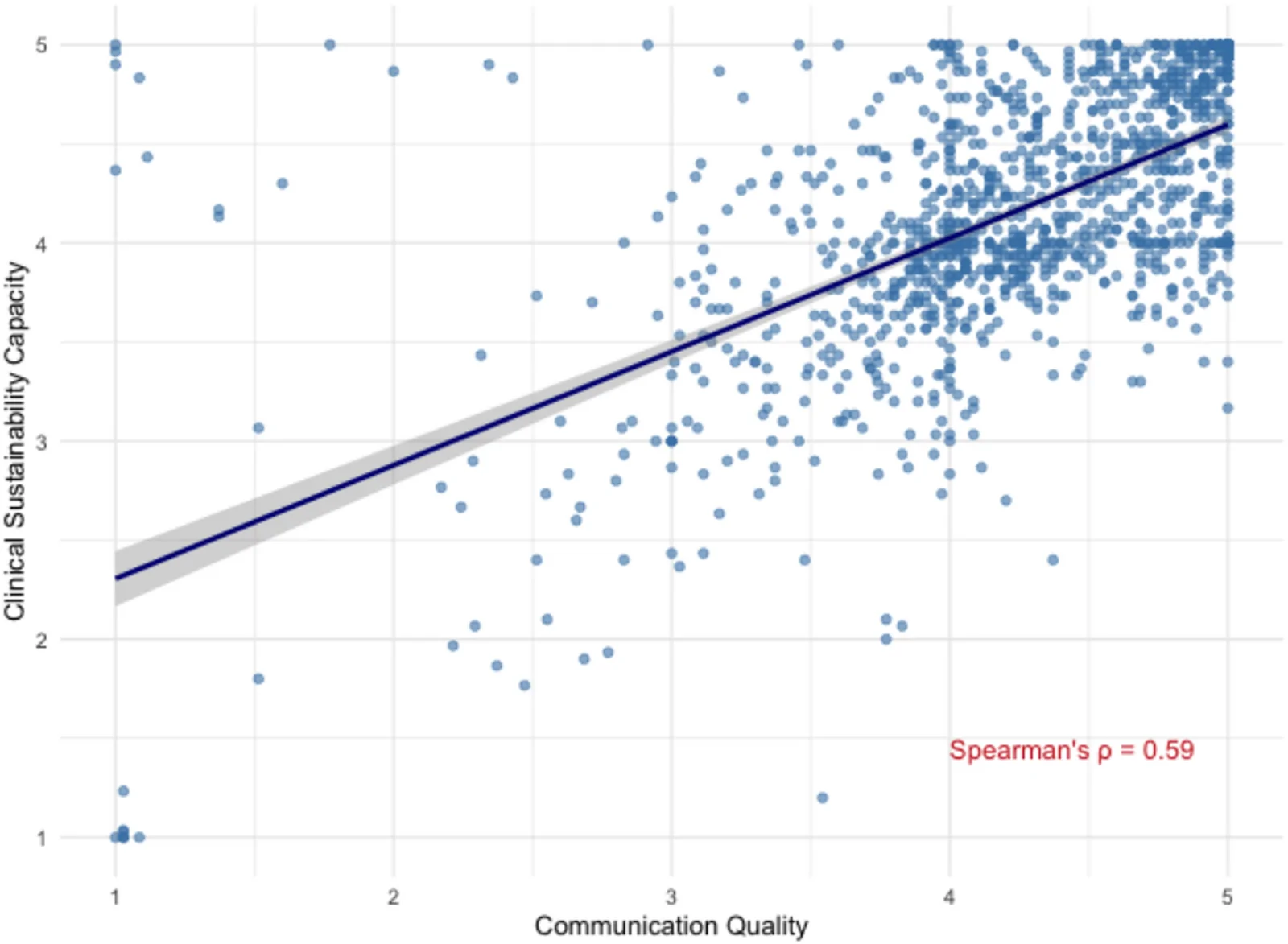
Correlation between communication quality (CritCom) and clinical sustainability capacity (CSAT) overall scores

Team communication quality (measured by CritCom) and clinical capacity to sustain PEWS (CSAT scores) were significantly and positively correlated (Spearman’s ρ = 0.59, *p* < 0.05), indicating a moderately strong monotonic relationship (Fig. 1). Scores on both scales clustered predominantly in the upper range, with a notable concentration in the upper-right quadrant of the scatterplot, reflecting a tendency for respondents who scored highly on one measure to also score highly on the other.

A null model was fit with hospital as the only grouping factor to assess the degree of clustering in CSAT scores. The ICC was 0.06, indicating that 6% of the variance in CSAT scores was attributable to between-hospital differences, supporting the use of a multilevel modeling approach. A linear mixed-effects model, using restricted maximum likelihood (REML), examined predictors of CSAT score while accounting for hospital-level clustering (Table 4). After controlling for other covariates, each one-unit increase in CritCom is associated with a 0.50-unit increase in CSAT score (β = 0.50, CI: 0.45–0.55). This association remained robust after adjustment for all covariates in the model, underscoring the independent relationship between communication quality and sustainability capacity.

**Table 4:** Mixed effects model results, significant p-values noted in bold

	Coef.	(95% CI)
Intercept	1.10	(0.14–2.06)
***CritCom Score (Communication Quality)***	***0.50***	***(0.45–0.55)***
*Gender (Reference: Male)*		
Gender: Female	0.03	(-0.05-0.12)
Gender: Other	-0.10	(-0.71-0.51)
*Implementation Position (Reference: Implementation Lead)*		
Position: Clinical Staff	*-0.03*	(-0.11-0.05)
Position: Hospital Administrator	*-0.16*	(-0.43-0.11)
***Position: Data Manager***	***-0.36***	***(-0.69 - -0.03)***
Position: Other	*-0.12*	(-0.30-0.05)
*Profession (Reference: Nurse)*		
**Profession: Physician**	**0.13**	**(0.06–0.21)**
Profession: Health Administration	0.11	(-0.08-0.30)
Position: Research/Data Manager	-0.07	(-0.52-0.35)
Profession: Other	-0.09	(-0.22-0.08)
*Clinical Location (Reference: Heme/Onc Unit)*		
***Role: Intensive Care Unit***	***0.05***	***(0.01–0.24)***
***Role: Non-Clinical***	***− 0.07***	***(0.07–0.55)***
Role: Emergency Department	− 0.46	(-0.13-0.32)
Role: Other	− 0.04	(-0.16-0.14)
*Years Experience (Reference: <1 Year)*		
Years: 1–5	-0.01	(-0.13-0.11)
Years: 6–10	-0.06	(-0.19-0.07)
Years: 11–15	-0.05	(-0.18-0.09)
Years: 16–20	0.00	(-0.14-0.15)
Years: 20+	0.02	(-0.12-0.17)
*Int. Importance (Reference: Not important)*		
Int. Importance: Neither important nor unimportant	0.22	(-0.42-0.85)
Int. Importance: Important	0.49	(-0.12-1.11)
Int. Importance: Very important	0.59	(-0.03-1.20)
*Intervention: Frequency of Delivery (reference: None of the time)*		
Int. Frequency: Some of the time	0.09	(-0.63-0.82)
Int. Frequency: Most of the time	0.19	(-0.52-0.89)
Int. Frequency: All of the time	0.34	(-0.36-1.04)
*Funding Structure (reference: Mix Private/Public)*		
Funding: Private	0.17	(-0.03-0.37)
Funding: Public	0.05	(-0.10-0.20)
*Organization Type (reference: General Hospital)*		
Site type: Oncology Adult/Peds	-0.04	(-0.20-0.13)
Site type: Pediatric multidisciplinary	0.05	(-0.08-0.18)
Site type: Pediatric Oncology	-0.05	(-0.30-0.20)
Site type: Women’s and Children’s Hospital	0.05	(-0.19-0.29)
*Hospital Annual Pediatric Oncology Diagnoses*	0.00	(0.00–0.00)
*Inpatient Bed Number*	0.00	(-0.01-0.0)

After adjustment for the other model covariates, participants working in the intensive care unit and in non-clinical areas reported lower CSAT scores than those working in oncology wards (*p* = 0.04 and 0.01, respectively). Additionally, physicians reported higher CSAT scores when compared to nurses (*p* < 0.01). No statistically significant differences in CSAT scores were observed by years of experience working at the hospital, frequency of PEWS delivery, or gender. Hospital characteristics were not significant in the multilevel model, including funding structure, organization type, annual volume of new pediatric cancer diagnoses, and number of inpatient beds.

To evaluate whether specific dimensions of communication quality were differentially associated with sustainability capacity, we conducted supplementary mixed-effects models that assessed CSAT scores separately for each of the six CritCom domains (actionability, clarity, tone, empowerment, collaboration, and leadership), adjusting for the same covariates as in the primary model. All six domains were independently, positively associated with CSAT score (*p* < 0.001 for each). However, model fit varied across domains: collaboration (AIC = 323.8) and empowerment (AIC = 459.7) showed the strongest associations with sustainability capacity, followed by tone (AIC = 626.0) and leadership (AIC = 802.8), while clarity (AIC = 815.8) and actionability (AIC = 1070.6) showed comparatively weaker, though still statistically significant, associations.

## Discussion

This study examined the relationship between team communication quality and clinical capacity to sustain PEWS, an evidence-based intervention to improve childhood cancer care, among resource-variable hospitals in Latin America and Spain. Findings demonstrate meaningful variation in both perceived team communication quality (measured by CritCom) and clinical sustainability capacity (measured by CSAT) across individual-level demographics and reveal a moderately strong positive association between communication quality and sustainability capacity. These findings suggest that communication quality is an important and under-considered determinant of sustainability capacity for clinical interventions across variable hospital settings.

Physicians and staff in PEWS leadership roles reported higher communication quality and sustainability capacity, a finding consistent with prior literature [9, 11, 18]. Differences in how physicians and nurses perceive team communication quality are well documented; a recurring theme is that nurses typically report lower satisfaction with communication patterns than their physician colleagues [19]. Staff in formal leadership roles, such those leading implementation teams, may also experience greater agency in intervention delivery and broader visibility into team coordination processes. Future sustainability research must engage clinical staff beyond leadership to ensure inclusion and perceptions from all levels and professions of clinicians involved in intervention use long-term. Efforts to strengthen sustainability may need to prioritize formal mechanisms for nurse engagement in implementation decision-making, rather than focusing solely on general communication skill-building. Systematic assessment of communication quality and clinical capacity from the perspective of all clinical staff, using tools validated multi-lingual such as CritCom and CSAT, can support identification of modifiable challenges to intervention sustainability.

The observed pattern of younger respondents reporting higher communication quality and sustainability capacity also warrants consideration. This finding may reflect generational differences in training and exposure to team-based care models. However, given the cross-sectional nature of this study, it is not possible to determine whether these differences reflect age itself, career stage, cohort effects, or unmeasured confounders. Future research should examine how perceptions of communication quality and sustainability capacity evolve across clinician career trajectories and how staff of differing clinical experience impact use and sustainability of evidence-based interventions in hospital settings. This pattern may also reflect structural rather than purely individual factors: newer staff may have been trained in institutions that have more recently adopted team-based care models that emphasize communication, while staff who have been around longer may continue to operate within more traditional hierarchies and workflows.

These findings situate communication quality as an important modifiable determinant of sustainability capacity in global pediatric oncology care. Poor communication quality can lead to worsened provider outcomes (burnout) and coordination of care, resulting in inferior patient outcomes [7]. Barriers to long-term sustainability of effective interventions often stem from competing priorities, lack of staff buy-in, and inconsistent engagement, all of which could be further challenged by poor communication [20]. While team communication has been extensively studied in relation to clinician-level outcomes, implementation science has largely treated communication as a transactional process focused on information exchange and has not adequately studied its role in enabling and sustaining complex interventions over time [21]. The positive association observed here between CritCom and CSAT extends prior literature by suggesting that team communication quality may shape the conditions under which evidence-based interventions are maintained over time. This is relevant, as health system improvement in resource-variable settings depends fundamentally on understanding the contexts in which care is delivered. Interprofessional relationships between healthcare staff shape not only the quality of clinical encounters but also the ability to adopt, adapt, and sustain clinical interventions over time. Similarly, communication quality does not arise in isolation; it is shaped by staffing levels, workload, organizational hierarchy, and resource availability, suggesting that efforts to strengthen communication may be most effective when paired with attention to these underlying structural conditions. This work highlights the urgent need to develop effective interventions to improve the quality of team communication in clinical care, not only to directly enhance patient and provider outcomes, but also to promote sustainability of evidence-based interventions.

Results of this study are consistent with other implementation theories and approaches, such as Normalization Process Theory (NPT) [22], which proposes that the sustainability of complex interventions depends on the collective action of clinical teams, which relies on communication across these professionals. These findings further raise the possibility of a cyclical, dynamic relationship: teams with stronger communication may be better positioned to sustain interventions like PEWS, and the ongoing practice of implementing structured intervention may reinforce and strengthen team communication over time, which in turn may further support intervention sustainability, and so forth. Conversely, organizational structures and processes that support implementation readiness and staff engagement, such as protected time for team meetings, leadership accessibility, clear role definition, and adequate staffing, may themselves foster the conditions under which effective communication develops. Future research should examine this dynamic empirically, including whether communication-focused implementation strategies improve sustainability capacity across clinical settings.

Supplementary domain-level analyses further suggest that not all dimensions of communication quality relate equally to sustainability capacity. While all six CritCom domains were independently associated with CSAT scores, collaboration and empowerment showed the strongest associations, while clarity and actionability showed comparatively weaker associations. This pattern suggests that, for this intervention, the relational and interpersonal qualities of team communication, such as whether staff feel respected, included, and able to influence team decisions, may matter more for sustained use of interventions than whether information is exchanged clearly or generates a specific action.

The association between communication quality and sustainability capacity observed in this study was robust after adjustment for hospital funding structure, organization type, oncologic patient volume, and number of inpatient beds, suggesting that this relationship was not dependent on structural or material resources. This is particularly meaningful in the context of resource-variable hospitals, where communication quality may represent a modifiable, low-cost lever for sustainability. Unlike structural interventions that require capital investment or workforce expansion, efforts to improve team communication are feasible across the full spectrum of hospital settings — from well-resourced academic centers to safety-net and rural hospitals that have historically faced barriers to implementing and sustaining evidence-based practices. These findings underscore the potential of communication-focused interventions to narrow gaps in sustainability across diverse contexts.

This study has several limitations. Data were collected from hospitals predominantly in a single geographic region (Latin America), which limits generalizability to other hospital settings providing childhood cancer care. Additionally, both CritCom and CSAT rely on self-reported, perception-based measures rather than observed team behaviors or objective clinical performance data; as such, responses may be subject to social desirability or common-method bias and may not fully correspond to actual communication practices or system-level functioning. While participating hospitals differed in hospital structure, resource-level, and other hospital characteristics, the outcomes observed in this study should be considered and evaluated in other regions. Additionally, the cross-sectional design precludes causal inference and primarily highlights that communication quality and sustainability capacity are associated. Without longitudinal data, it is not possible to assess how these variables change over time, which is a notable gap, given that implementation and sustainability are inherently time-dependent processes. Future studies should focus on the directionality and mechanisms of this relationship, ideally using longitudinal designs that track communication and sustainability capacity across implementation phases. Future research would also benefit from incorporating clinical outcomes associated with PEWS implementation, such as childhood cancer mortality, unplanned PICU transfers, or hospital readmissions, to determine whether perceived communication quality and sustainability capacity correspond to measurable improvements in patient care.

## Conclusion

This study presented a multi-country assessment of individuals implementing PEWS across a variety of resources and revealed that perceptions of team communication quality is associated with sustainability capacity, with meaningful variability by professional role and role in the intervention. These findings describe communication quality as a modifiable determinant of sustainability that warrants further examination and targeted intervention. Multilevel assessment of this relationship is necessary to move toward actionable targets for sustainability strategies that promote long-term use of effective interventions to reduce the global burden of childhood cancer.

## Supplementary Information

Below is the link to the electronic supplementary material.

Supplementary Material 1

Supplementary Material 2

## Data Availability

De-identified individual participant data that underlie the results reported in this Article, together with a data dictionary, will be available upon reasonable request to the corresponding author.
