# Supplementary material for: Communication quality shapes the sustainability of pediatric early warning system
Source: BMC Health Serv Res. 2026 Aug 1;26:1504. doi: 10.1186/s12913-026-15265-9 (PMC13726181; doi:10.1186/s12913-026-15265-9)
Supplement: Supplementary file 1 [file 12913_2026_15265_MOESM1_ESM.pdf]

Actionable: Allows for getting the job done (when and what). Communication that is timely, relevant, and contains the necessary information to act.

	Almost Never	Rarely	Sometimes	Often	Almost Always
1. Staff in my unit communicate patient information in a timely manner.	○	○	○	○	○
2. When a patient's status changes, staff communicate all relevant information to make management decisions.	○	○	○	○	○
3. During shift changes, staff exchange all essential patient information.	○	○	○	○	○
4. Staff communicate complete information when transferring a patient with deterioration from one unit to another.	○	○	○	○	○
5. I can reach someone easily when I need to communicate about a deteriorating patient.	○	○	○	○	○

Clarity: Content of communication that is clear, complete, structured, and communicates a shared mental model.

	Almost Never	Rarely	Sometimes	Often	Almost Always
1. Staff verbally confirm information that they receive from one another.	○	○	○	○	○
2. Our team uses standard medical language to communicate with other staff about a deteriorating patient's status.	○	○	○	○	○
3. Staff seek clarification about unclear information.	○	○	○	○	○
4. After talking about a patient with deterioration, staff have the same understanding about the next steps in management.	○	○	○	○	○
5. Our team communicates clearly.	○	○	○	○	○

Tone: Understanding positive and negative communication styles and wording. This includes non-verbal communication and being ignored.

	Almost Never	Rarely	Sometimes	Often	Almost Always
1. Communication between staff in my unit has a positive tone.	○	○	○	○	○
2. In my unit, we talk about errors without placing blame.	○	○	○	○	○
3. Staff demonstrate active listening with other team members.	○	○	○	○	○
4. Staff in my unit use approachable non-verbal communication (body language, facial expression).	○	○	○	○	○
5. Constructive criticism between staff is delivered in a way that promotes positive interactions.	○	○	○	○	○

Empowerment: This assesses a team member's ability and comfort to proactively evaluate patients, make decisions, speak up, and escalate concerns without fear of consequences.

	Almost Never	Rarely	Sometimes	Often	Almost Always
1. I feel empowered to communicate freely with others on my team.	○	○	○	○	○
2. It is easy for staff in my unit to ask questions when there is something that they do not understand.	○	○	○	○	○
3. I feel empowered to advocate for the care plan that I believe is appropriate for my patient.	○	○	○	○	○
4. Staff in my unit are comfortable questioning the decisions or actions of those with more authority.	○	○	○	○	○
5. Staff in my unit are comfortable calling for help for a patient with deterioration.	○	○	○	○	○

Collaboration and Teamwork: Interdisciplinary collaboration between members in patient's care team. This includes working together, mutual respect, and role clarity.

	Almost Never	Rarely	Sometimes	Often	Almost Always
1. In my unit, there is good collaboration between nurses and physicians.	○	○	○	○	○
2. I feel supported when discussing challenging patient care issues with other staff in my unit.	○	○	○	○	○
3. Staff in my unit work well together as a team.	○	○	○	○	○
4. Constructive criticism is welcome from any member of the team.	○	○	○	○	○
5. Decisions regarding patient care are made collaboratively with input from all relevant disciplines (oncology, surgery, ICU, etc.).	○	○	○	○	○

Leadership: Assessing influences of organizational leadership and reporting structures that impede or facilitate communication.

	Almost Never	Rarely	Sometimes	Often	Almost Always
1. Unit leadership believes interdisciplinary communication is important.	○	○	○	○	○
2. Unit leadership is open to hearing patient safety concerns from staff.	○	○	○	○	○
3. Unit leadership encourages nurses to take initiative.	○	○	○	○	○
4. There is a clear chain of command (leadership structure) in my unit.	○	○	○	○	○
5. Unit leadership intervene if staff act in a way that impedes effective communication.	○	○	○	○	○
