# Supplementary material for: Communication quality shapes the sustainability of pediatric early warning system
Source: BMC Health Serv Res. 2026 Aug 1;26:1504. doi: 10.1186/s12913-026-15265-9 (PMC13726181; doi:10.1186/s12913-026-15265-9)
Supplement: Supplementary file 2 [file 12913_2026_15265_MOESM2_ESM.pdf]

Clinical Sustainability Assessment Tool v2

What is clinical sustainability capacity?

We define clinical sustainability capacity as the ability of an organization to maintain structured clinical care practices over time and to evolve and adapt these practices in response to new information.

Why is clinical sustainability capacity important?

Without sustaining effective practices over time, we risk not being able to see the full return on our nation's investment in clinical and translational science. Successful implementation of new practices in clinical or healthcare settings is affected by a number of organizational, financial, regulatory, and political factors. To maintain these benefits, clinical settings and healthcare organizations must support these clinical practices in a number of ways. With knowledge of these critical factors, interested parties can build capacity for sustainability of a clinical practice and position their efforts for long term success.

What is the purpose of this tool?

This tool will help structure an assessment of your group's current capacity for sustainability across a range of specific organizational and contextual factors. Your responses will identify sustainability strengths and challenges. You can then use results to guide sustainability action planning for your clinical practice.

Helpful definitions

The Clinical Sustainability Assessment Tool can be used in a wide variety of clinical practice settings. Before starting the assessment, you should identify the specific clinical practice you will be assessing.

Below are a few definitions of terms that are frequently used throughout the tool.

- **Practice** refers to the set of formal organized activities that you want to sustain over time. Such activities could occur in a variety of clinical settings.
- **Organization** encompasses all the parent organizations or agencies in which the practice is housed. Depending on the practice, the organization may refer to a health center, a hospital, etc.
- **Community** refers to the interested parties who may benefit from or who may guide the practice. This could include clinical staff, leadership, care recipients and their families, etc. Community does not refer to a specific town or neighborhood.

Copyright 2023. The Clinical Sustainability Assessment Tool is a copyrighted instrument of Washington University, St Louis, MO. All rights reserved. If you would like more information about the framework or our sustainability assessment tool, visit http://www.sustaintool.org.

The next question is to serve as a reference point for the following survey. Some examples of clinical practices are: antibiotic stewardship, a new surgical procedure, electronic medical record order sets, or hand hygiene.

The name of the practice or set of activities I am assessing is:

____

In the following questions, you will rate your practice across a range of specific factors that affect sustainability. Please respond to as many items as possible. If you truly feel you are not able to answer an item, you may select "NA." **For each statement, circle the number that best indicates the extent to which your practice has or does the following things.**

Engaged Staff & Leadership: Having supportive frontline staff and management within the organization

	To little or no extent						To a very great extent	Not able to answer
1. The practice engages leadership and staff throughout the process.	1	2	3	4	5	6	7	NA
2. Clinical champions of the practice are recognized and respected.	1	2	3	4	5	6	7	NA
3. The practice has engaged, ongoing champions.	1	2	3	4	5	6	7	NA
4. The practice has a leadership team made of multiprofessional partnerships.	1	2	3	4	5	6	7	NA
5. The practice has team-based collaboration and infrastructure.	1	2	3	4	5	6	7	NA

For each statement, circle the number that best indicates the extent to which your practice has or does the following things.

Engaged Partners: Having external support and engagement for the practice.

	To little or no extent						To a very great extent	Not able to answer
1. The practice engages the patient and family members as partners.	1	2	3	4	5	6	7	NA
2. There is respect for all partners involved in the practice.	1	2	3	4	5	6	7	NA
3. The practice is valued by a diverse set of interested parties.	1	2	3	4	5	6	7	NA
4. The practice engages other medical teams and community partnerships as appropriate.	1	2	3	4	5	6	7	NA
5. The practice team has the ability to respond to feedback about the practice.	1	2	3	4	5	6	7	NA

Organizational Readiness: Having the internal support and resources needed to effectively manage the practice

	To little or no extent						To a very great extent	Not able to answer
1. Organizational systems are in place to support the various practice needs.	1	2	3	4	5	6	7	NA
2. The practice fits in well with the culture of the team.	1	2	3	4	5	6	7	NA
3. The practice has feasible and sufficient resources (e.g., time, space, funding) to achieve its goals.	1	2	3	4	5	6	7	NA
4. The practice has adequate staff to achieve its goals.	1	2	3	4	5	6	7	NA
5. The practice is well integrated into the operations of the organization	1	2	3	4	5	6	7	NA

For each statement, circle the number that best indicates the extent to which your practice has or does the following things.

Workflow Integration: Designing the practice to fit into existing practices and technologies

	To little or no extent						To a very great extent	Not able to answer
1. The practice is built into the clinical workflow.	1	2	3	4	5	6	7	NA
2. The practice is easy for clinicians to use.	1	2	3	4	5	6	7	NA
3. The practice integrates well with established clinical practices.	1	2	3	4	5	6	7	NA
4. The practice aligns well with other clinical systems (e.g., EMR).	1	2	3	4	5	6	7	NA
5. The practice is designed to be used consistently.	1	2	3	4	5	6	7	NA

Implementation & Training: Using processes that guide the direction, goals and strategies of the practice

	To little or no extent						To a very great extent	Not able to answer
1. The practice clearly outlines roles and responsibilities for all staff.	1	2	3	4	5	6	7	NA
2. The reason for the practice is clearly communicated to and understood by all staff.	1	2	3	4	5	6	7	NA
3. Staff receive ongoing coaching, feedback, and training.	1	2	3	4	5	6	7	NA
4. Practice implementation is guided by feedback.	1	2	3	4	5	6	7	NA
5. The practice has ongoing education across professions.	1	2	3	4	5	6	7	NA

For each statement, circle the number that best indicates the extent to which your practice has or does the following things.

Monitoring & Evaluation: Assessing the practice to inform planning and document results

	To little or no extent						To a very great extent	Not able to answer
1. The practice has measurable process components, outcomes, and metrics.	1	2	3	4	5	6	7	NA
2. Evaluation and monitoring of the practice are reviewed on a consistent basis.	1	2	3	4	5	6	7	NA
3. The practice has clear documentation to guide process and outcome evaluation.	1	2	3	4	5	6	7	NA
4. Practice monitoring, evaluation, and outcomes data are routinely reported to the clinical care team.	1	2	3	4	5	6	7	NA
5. The practice process components, outcomes, and metrics are easily assessed and audited.	1	2	3	4	5	6	7	NA

Outcomes & Effectiveness: Understanding and measuring practice outcomes and impact

	To little or no extent						To a very great extent	Not able to answer
1. The practice has evidence of beneficial outcomes.	1	2	3	4	5	6	7	NA
2. The practice is associated with improvement in patient outcomes that are clinically meaningful.	1	2	3	4	5	6	7	NA
3. The practice is clearly linked to positive health or clinical outcomes.	1	2	3	4	5	6	7	NA
4. The practice is cost-effective.	1	2	3	4	5	6	7	NA
5. The practice has clear advantages over alternatives.	1	2	3	4	5	6	7	NA

The *Clinical Sustainability Assessment Tool* is a copyrighted instrument of Washington University, St Louis MO. All rights reserved. This work is licensed under a Creative Commons Attribution-NonCommercial- ShareAlike License. If you modify this tool, please notify the Center for Public Health Systems Science. By using the *Clinical Sustainability Assessment Tool* you understand and agree to these terms of use and agree that Washington University bears no responsibility to you or any third party for the consequences of your use of the tool. If you would like more information about how to use this tool with your program or would like to learn about our sustainability workshops and webinars, visit http://www.sustaintool.org. 2023

Clinical Sustainability Assessment Tool v2

Rating Instructions

Once you have completed the Clinical Sustainability Assessment Tool, transfer your responses to this rating sheet to calculate your average scores. Please record the score for each item (1-7), or write "NA" if you were not able to answer.

		DOMAIN						
	ITEM	Engaged Staff & Leadership	Engaged Partners	Organizational Readiness	Workflow Integration	Implementation & Training	Monitoring & Evaluation	Outcomes & Effectiveness
	1.							
	2.							
	3.							
	4.							
	5.							
Add up your scores in each column. Exclude 'NA'	**Domain Total:**							
Divide the domain total by the total number of items with a score. Exclude 'NA'	**Average Score for Domain:**							
Average together all the domain scores	**Overall Score:**							

Use these results to guide sustainability action planning for your clinical practice. The domains with lower average scores indicate areas where your practice's capacity for sustainability could be improved.

Copyright 2023. The *Clinical Sustainability Assessment Tool* is a copyrighted instrument of Washington University, St Louis, MO. All rights reserved. If you would like more information about the sustainability framework or the tool, visit http://www.sustaintool.org.

Center for Public Health Systems Science

WashU Public Health
